# Functionalized Copper Nanoparticles with Gold Nanoclusters: Part I. Highly Selective Electrosynthesis of Hydrogen Peroxide

**DOI:** 10.1021/acsomega.3c03665

**Published:** 2023-09-22

**Authors:** Kun Luo, Ya Li, Tong Liu, Xiangqun Zhuge, Etelka Chung, Andrew R. Timms, Simon P. Graham, Guogang Ren

**Affiliations:** † School of Materials Science and Engineering, Changzhou University, Changzhou 213164, P. R. China; ‡ University of Hertfordshire, Hatfield, Hertfordshire AL10 9AB, U.K.; § The Pirbright Institute, Ash Road, Pirbright, Woking GU24 0NF, U.K.

## Abstract

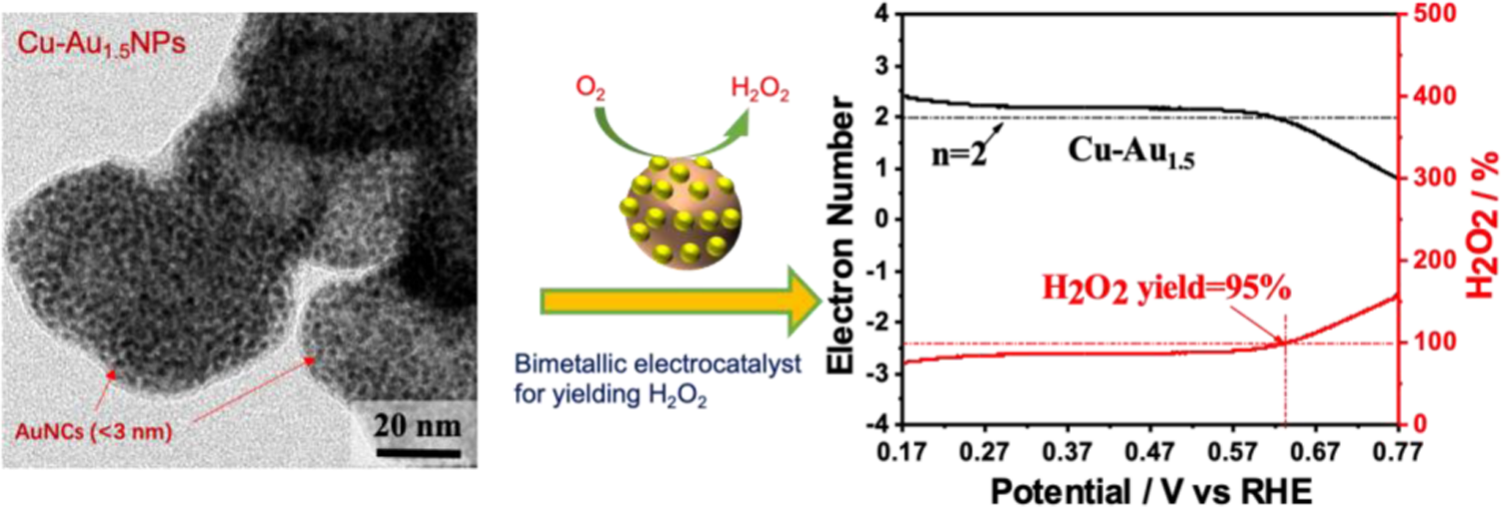

Copper nanoparticles (CuNPs) and gold nanoclusters (AuNCs) show a high catalytic performance in generating hydrogen peroxide (H_2_O_2_), a property that can be exploited to kill disease-causing microbes and to carry carbon-free energy. Some combinations of NPs/NCs can generate synergistic effects to produce stronger antiseptics, such as H_2_O_2_ or other reactive oxygen species (ROS). Herein, we demonstrate a novel facile AuNC surface decoration method on the surfaces of CuNPs using galvanic displacement. The Cu–Au bimetallic NPs presented a high selective production of H_2_O_2_ via a two-electron (2e^–^) oxygen reduction reaction (ORR). Their physicochemical analyses were conducted by scanning electron microscopy (SEM), transmitting electron microscopy (TEM), X-ray diffraction (XRD), and X-ray photoelectron spectroscopy (XPS). With the optimized Cu–Au_1.5_NPs showing their particle sizes averaged in 53.8 nm, their electrochemical analysis indicated that the pristine AuNC structure exhibited the highest 2e^–^ selectivity in ORR, the CuNPs presented the weakest 2e^–^ selectivity, and the optimized Cu–Au_1.5_NPs exhibited a high 2e^–^ selectivity of 95% for H_2_O_2_ production, along with excellent catalytic activity and durability. The optimized Cu–Au_1.5_NPs demonstrated a novel pathway to balance the cost and catalytic performance through the appropriate combination of metal NPs/NCs.

## 1 Introduction

Hydrogen peroxide (H_2_O_2_) is a green oxidizing agent, which has been used broadly in the modern chemical industry,^1^ antibiotics, and antivirals,^2^ as well as environmental remediation such as disinfection or decontamination.^3,4^ H_2_O_2_ is also a promising carbon-free energy carrier,^5^ which can be readily stored and used as needed to generate electricity by using fuel cells. According to Global Market Insights Inc., the H_2_O_2_ market size is forecast to exceed $6.2 billion by 2026.^6^ Currently, H_2_O_2_ is mainly produced by the indirect anthraquinone method (AQ),^7^ which involves multiple redox reactions and requires expensive platinum (Pd)-based hydrogenation catalysts. The direct synthesis of H_2_O_2_ from H_2_–O_2_ mixtures is still immature and has an explosion risk.^8^ Therefore, the oxygen reduction reaction (ORR) through a two-electron (2e^–^) reduction process from O_2_ is a cost-effective method using air as an abundant resource,^9^ which is also safer and cleaner.

Reactive oxygen species (ROS) can induce oxidative stress in bacteria and viruses. ROS exhibits different dynamics and activity against pathogens, which covers the actions from the superoxide radical (O_2_^–^), the hydroxyl radical (^•^OH), H_2_O_2_, and singlet oxygen (^1^O_2_). ROS production is responsible for the antibacterial and antiviral mechanisms of the NPs/NCs.^10^ For example, copper oxide (CuO) NPs can produce all four types of ROS,^1,2,11,12^ and studies show that ^•^OH and O_2_^–^ can oxidize enzymes and lead to acute microbial death. The main causes of ROS production are attributed to the restructuring, defect sites, and oxygen vacancies in the crystals of nanoparticles.^13,14^

Noble metals such as platinum (Pt) and Pd interact strongly with O_2_, which leads to complete water (or OH^–^) reduction via 4e^–^ transfer.^15,16^ When Pt or Pd are alloyed with other metals, such as mercury (Hg) or gold (Au), the 2e^–^ ORR selectivity was reported to be improved.^17^ Au nanoparticles (AuNPs) are also known experimentally to selectively reduce O_2_ to H_2_O_2_ with higher faradic efficiency;^18^ however, activating O_2_ to form the key intermediate OOH* remains weaker than Pt, owing to the weak interaction of the Au surface with O_2_. This can be altered by the choice of substrates, crystallographic orientation, particle size, capping ligands, and the pH values of electrolytes.^19−22^ As a nonprecious metal, CuNPs also possess good ORR catalytic activity,^3,4,23,24^ especially when dispersed at an atomic or quantum level.^19,25^ However, the electron transfer number of pure Cu varies from 2e^–^ to 4e^–^ with the increase of overpotential.^18^

To reduce the amount of precious metals required without compromising catalytic performance, bimetallic catalysts have been developed and attracted interest.^26,27^ In the material design, the precious metal played an active site, while the material enhanced the catalytic role by increasing the surface area and exerting a synergistic effect by the catalyst–substrate interaction. Sarkar et al.^28^ reported the synthesis of Cu–Pt bimetallic nanoparticles, which exhibited higher ORR activity compared to the commercial pure Pt catalyst. Zhang et al.^29^ synthesized Pd–Pt bimetallic catalysts for ORR by a galvanic displacement reaction between Pd atoms and Pt cations, which showed 4 times higher activity under the same Pt mass and improved durability upon potential cycling time than that of the commercial Pt/C catalyst.

In this study, we demonstrate the bimetallic catalytic functionalization of nonprecious CuNPs with AuNCs by using a galvanic displacement reaction, which led to the raspberry-like Cu–AuNPs with a high 2e^–^ ORR selectivity of 95% for H_2_O_2_ production. The distinctive combination of CuNPs/AuNCs allows a cost-effective balance to be reached against the catalyst selection and their catalytic performance for producing H_2_O_2_, which can be readily beneficial to healthcare and energy-storage applications.

## 2 Materials and Experimental Methodology

### 2.1 Materials and Chemicals

Chloroauric acid (HAuCl_4_, Au content 48–50%, Shanghai McLean Technology Biochemical Co., Ltd.), poly(vinylpyrrolidone) (PVP, Shanghai Wokai Chemical Reagent Co., Ltd.), and mercaptosuccinic acid (MSA, C_4_H_6_O_4_S, Shanghai Aladdin Biochemical Technology Co., Ltd., 98%) were used as received. Copper sulfate (CuSO_4_, ≥99.0%), potassium hydroxide (KOH, ≥85.0%), sodium borohydride (NaBH_4_, ≥98.0%), and ethylenediaminetetraacetic acid (EDTA, C_10_H_16_N_2_O_8_, ≥99.5%) were all with analytical purity, obtained from Sinopharm Group Chemical Reagent Co., and they were all used without further treatment.

### 2.2 Synthesis of Cu–Au Bimetallic Electrocatalysts

#### 2.2.1 CuNP Preparation

CuNPs were first synthesized by the reduction process of CuSO_4_ in a NaBH_4_ water solution. Overall, 22.4 g of KOH and 2.7 g of NaBH_4_ were dissolved in a conical flask containing 200 mL of deionized water. Then, 8 g of EDTA and 8 g of PVP were added to the solution. The solution in the flask was stirred in a water bath at 40 °C, followed by a dropwise injection of 200 mL of a 0.8 M CuSO_4_ water solution at a rate of 50 drops/min. The reaction was carried out under magnetic stirring for 30 min, and then the solid in suspension was separated by centrifugation at 12 000 rpm (producing 13 820 g-force). The precipitate was rinsed 3 times with deionized water, followed by rinsing with anhydrous ethanol 3 times. Finally, the suspension was freeze-dried and yielded black CuNP powders, as shown in Figure S1.

#### 2.2.2 Cu–AuNP Preparation

Cu–AuNP bimetallic catalysts were synthesized by decorating the precursor surfaces of CuNPs using AuNCs through a simple galvanic displacement reaction at the CuNP’s surfaces as follows:
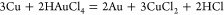
1where 28.8 mg of the as-synthesized CuNPs was dispersed in 10 mL of an ethanol solution by magnetic stirring at ambient temperature, followed by additions of 10 mL of 6.75 mM HAuCl_4_ and 10 mL of 13.5 mM of MSA (capping agent or capping molecules). After reacting for 2 h, the solid in the suspension was separated by centrifuging at 12 000 rpm (13 820 g-force). The precipitate was rinsed 3 times with deionized water and then washed 3 times with anhydrous ethanol. After freeze-drying, the brown Cu–AuNPs were obtained (as shown in Figure S1), marked as Cu–Au_1.5_. As references for compositional optimization, Cu–Au_1_, Cu–Au_2_, and pure AuNCs were also made, and methods are listed below:

(1) Cu–Au_1_: A solution containing 10 mL of 4.5 mM HAuCl_4_ and 10 mL of 9.0 mM MSA was employed to treat 28.8 mg of as-synthesized CuNPs that were dispersed in 10 mL of an ethanol solution, resulting in the powder named as Cu–Au_1_.
(2) Cu–Au_2_: When the solution contained 10 mL of a 9.0 mM HAuCl_4_ solution and 10 mL of 18.0 mM MSA in the galvanic displacement reaction, we obtained the sample noted as Cu–Au_2_.
(3) AuNCs: 5 mL of deionized water, 500 μL of 25 mM HAuCl_4_, and 500 μL of 50 mM MSA were mixed by magnetic stirring, and then 1 mL of 50 mM NaBH_4_ was dropwise injected. The reaction was maintained for 1 h, and then the resulting suspension was purified in a dialysis bag with deionized water, where the deionized water in the bath for dialysis was renewed every day. After 7-day dialysis, the suspension was freeze-dried and the pristine AuNCs were obtained.

### 2.3 Characterizations

The morphology and composition of the as-synthesized nanomaterials were characterized using a field-emission desktop scanning electron microscope (Phenom LE, Thermo Fisher Scientific) and a high-resolution transmission electron microscope (JEM-2100, JEOL). An X-ray diffractometer (XRD, D/max 2500PC, Rigaku) was used to examine the morphological and structural properties of all nanoparticles. An X-ray photoelectron spectroscope (XPS, ESCALAB 250Xi, Thermo Fisher Scientific) was also employed to provide accurate elemental compositions of the obtained nanomaterials.

The validation of the catalytic properties of these nanomaterials was performed using an electrochemical workstation (CHI760, Shanghai Chenhua Instrument Co., Ltd.). As shown in Figure S2, a three-electrode system was employed in the analysis within the workstation platform in which a glassy carbon (GC) electrode, a Ag/AgCl electrode, and a Pt wire electrode were used as an integrated working system composing reference and counter electrodes, respectively. Prior to use, the GC working electrode was modified by the nanoparticle slurry, where 4 mg of the as-prepared nanoparticles was dispersed into a vial with 800 μL of deionized water, 200 μL of ethanol, and 100 μL of a 5% Nafion solution under ultrasonic stirring for 30 min, as shown in Figure S2. In the cyclic voltammetry (CV), linear sweep voltammetry (LSV), and rotating disk electrode (RDE) analyses, the glassy carbon disk electrode (*d* = 3 mm, *S* = 0.07 cm^2^) was modified with 6 μL of the nanoparticle slurry, which was then dried at room temperature.

In the rotating ring-disk electrode (RRDE, ALS RRDE-3A, BAS Company) analysis, the working electrode consisted of a glassy carbon disk (*d* = 4 mm, *S*_GC_ = 0.126 cm^2^) and a Pt ring (*S*_Pt_ = 0.189 cm^2^). In this case, the glassy carbon disk was modified with 8 μL of the nanoparticle slurry, while the Pt ring was left free of modification and then allowed to dry at ambient temperature before use.

## 3 Results and Discussion

### 3.1 Morphology and Structure of Cu–AuNPs

**Figure 1:**
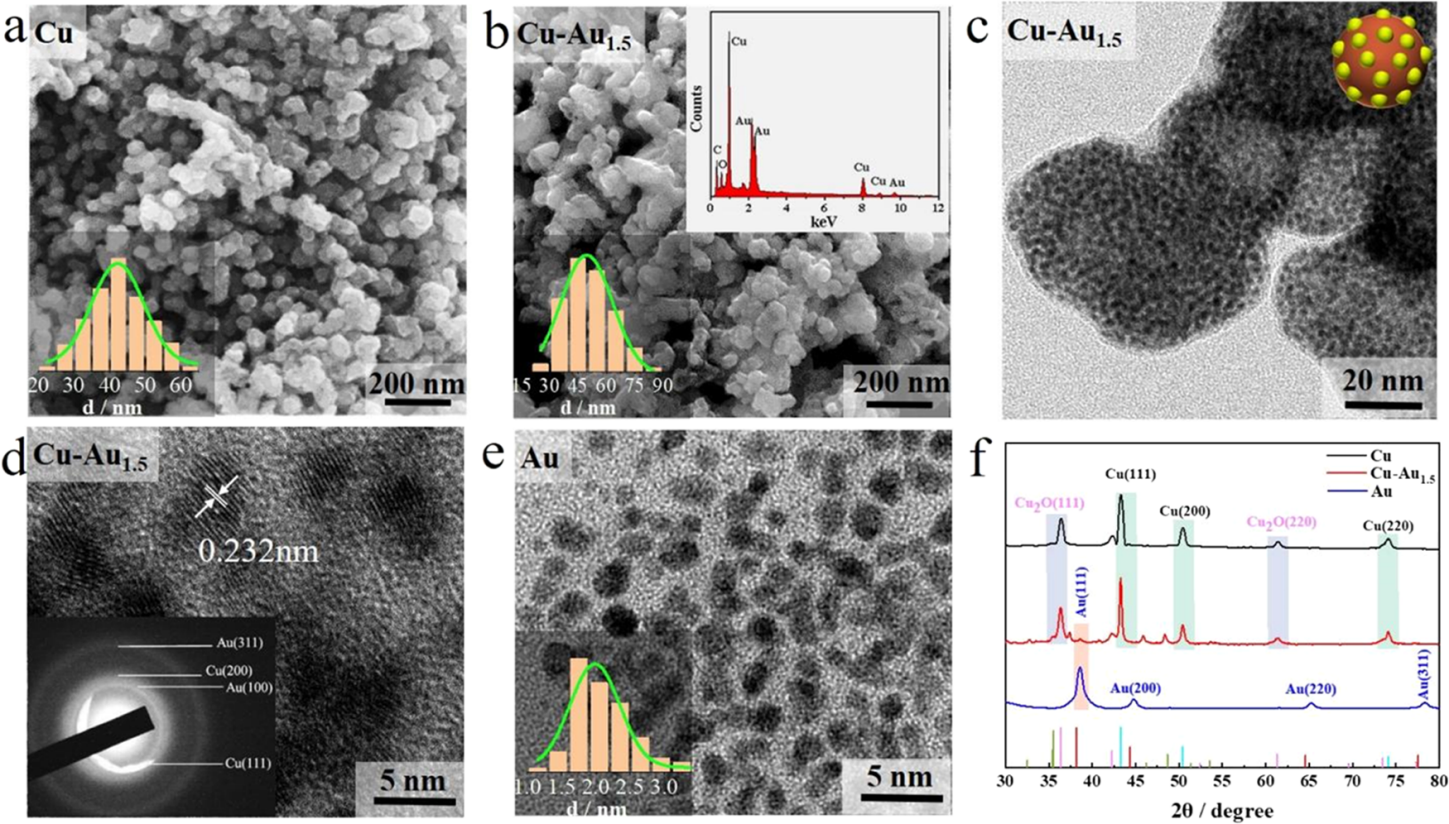
Characterization of CuNPs, Cu–Au_1.5_NPs, and AuNCs: (a) SEM image of the CuNPs (inset: size distribution), (b) SEM image of the Cu–Au_1.5_NPs: upper inset: EDS analysis and lower inset: size distribution, (c) TEM (c, inset: a conceptual illustration of raspberry structure), (d) HRTEM images of the Cu–Au_1.5_NPs (inset: SEAD analysis), (e) TEM image of AuNCs (inset: size distribution <2–3 nm), and (f) XRD patterns of the CuNPs, Cu–Au_1.5_NPs, and AuNCs.

The SEM images in Figure 1a,b display the morphology of the as-synthesized CuNPs and Cu–Au_1.5_NPs, respectively. Their average diameters are measured as 48.8 ± 4.8 nm (N = 245; CuNP) and 53.8 ± 6.5 nm (*N* = 240; Cu–Au_1.5_NPs), as shown in the lower insets, with the AuNC-capped CuNPs being slightly larger than the pure CuNPs. Energy dispersive spectroscopy (EDS) in the upper inset of Figure 1b confirms the presence of Au. Figure 1c shows a TEM image of the Cu–Au_1.5_NPs, where the black spots in the image represent the AuNCs capped on the CuNP substrates in the form of raspberry-like structure (see the conceptual illustration in the inset). These have been shown in the detailed high-resolution transmission electronic microscopic (HRTEM) micrograph in Figure 1d, illustrating the crystal plane spacing of the AuNCs, which is measured as 0.232 nm, corresponding to the Au(111) crystal plane. The selected area electron diffraction pattern in the inset manifests the Au(110), Au(311), Cu(111), and Cu(200) crystal planes, confirming the coexistence of Cu and Au in the as-synthesized Cu–Au_1.5_NPs. As a reference, Figure 1e shows the TEM image of the pure AuNCs, where the average size is detected as 2.8 ± 0.4 nm (*N* = 232), as depicted in the inset. Figure S3 shows the HRTEM analysis of the pure AuNCs, in which the (111) and (200) facets of Au crystals can be observed.

Figure 1f illustrates the XRD patterns of the Cu, Cu–Au_1.5_, and AuNCs. Three peaks at 43.2, 50.4, and 74.1° are seen in the pattern of CuNPs (black curve), corresponding to the Cu (111), (200), and (220) faces (JCPDF No. 04-0836). In addition, three more peaks are also observed at 29.5, 42.2, and 61.3°, attributed to the (111), (200), and (220) crystal planes of Cu_2_O (JCPDF No. 05-0667), respectively, indicating the oxidation of Cu metal. The red curve is the spectrum of the Cu–Au_1.5_NPs, which illuminates four characteristic peaks at 38.1, 44.3, 64.5, and 77.5°, respectively, assigned to the (111), (200), (220), and (311) planes of Au (JCPDF No. 89-3679). The peak at 29.5° indicates the (111) facet of Cu_2_O. The blue line displays three peaks at 38.1, 44.4, and 64.6°, attributed to Au (111), Au (200), and Au (220) faces of the AuNCs.

**Figure 2:**
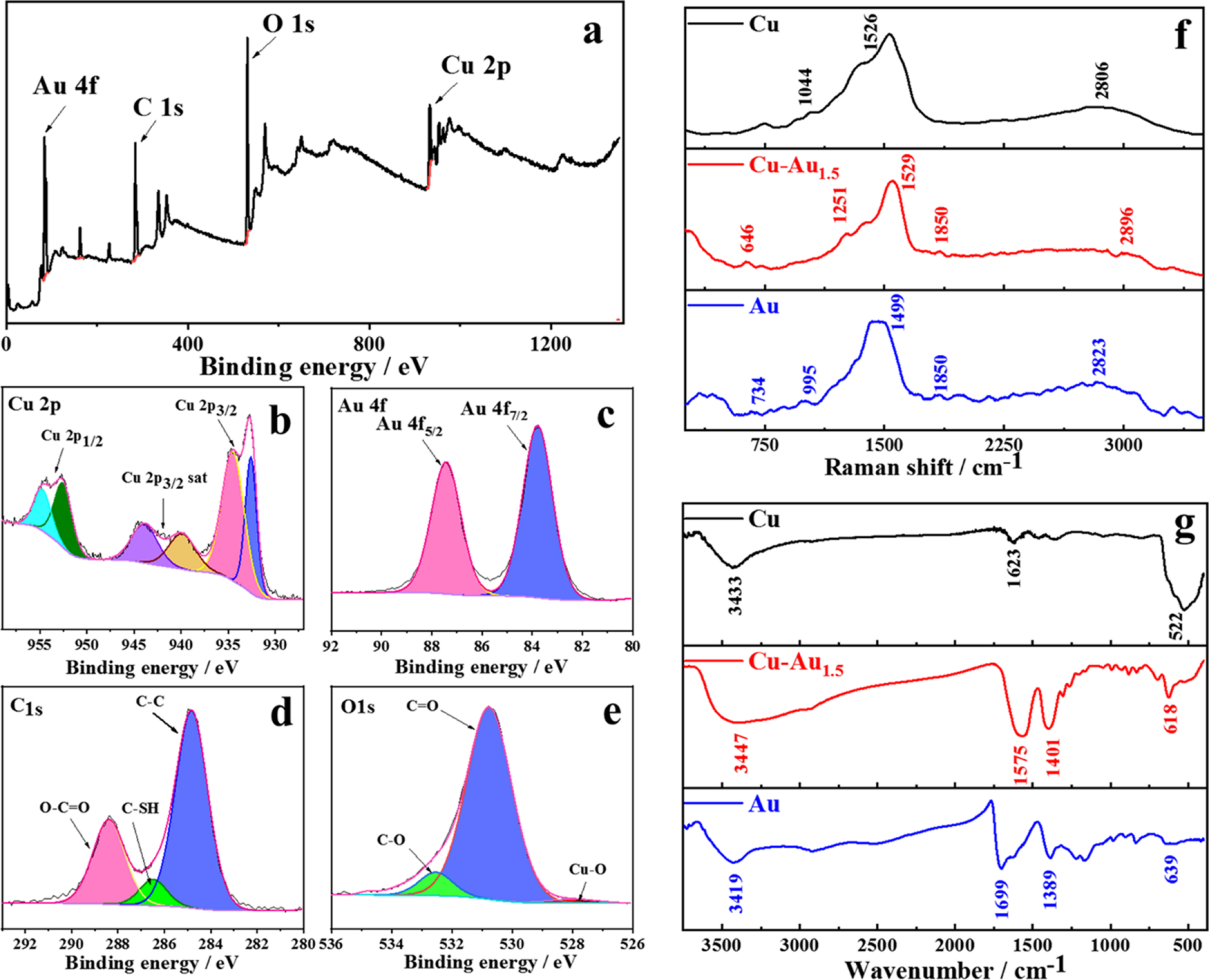
Chemical and compositional information on the Cu–Au_1.5_NPs obtained by X-ray photoelectron spectroscopy (XPS) shown as the XPS total spectrum (a) and fitting of the resolved Cu 2p (b), Au 4f as shown in (c), C 1s as shown in (d), and O 1s as shown in (e). The signals of the Cu–Au_1.5_NPs from Raman spectrum are shown in (f) and (g) where FTIR analyses of the CuNPs, Cu–Au_1.5_NPs, and AuNCs are displayed.

The as-synthesized Cu–Au_1.5_NPs were investigated by X-ray photoelectron spectroscopy (XPS) to gain their chemical and compositional information. As shown in Figure 2a, four peaks assigned to Cu 2p, Au 4f, O 1s, and C 1s displayed overall XPS absorptions. The high-resolution spectra of the Cu 2p electrons are shown in Figure 2b. Deconvolution yields two peaks at 932.6 and 934.4 eV for the Cu–Au_1.5_NPs sample, which can be ascribed to the 2p_3/2_ electrons of Cu/Cu^+^ and Cu^2+^,^30^ respectively. The weak satellite peaks at 939.4 and 943.5 eV correspond to Cu^2+^. Small amounts of Cu_2_O on the surface could be oxidized to CuO and/or Cu(OH)_2_ species when the surface is exposed to air with humidity.^31^ Owing to the different detection sensitivity and detection depth between XPS and XRD, Cu^2+^ can be detected by XPS in Cu_2_O, while CuO and Cu(OH)_2_ cannot be detected by XRD. For the signals of Cu 2p_1/2_, the peaks at 952.6 and 954.5 eV are assigned to Cu. Furthermore, the peak difference between Cu 2p_3/2_ (932.6 eV) and Cu 2p_1/2_ (952.6 eV) is 20 eV, suggesting that Cu^2+^ is present^32^ and is consistent with the results of XRD. Fitting of the resolved Au_4f_ spectrum in Figure 2c manifests a pair of peaks at 83.8 and 87.4 eV, corresponding to Au 4f_7/2_ and Au 4f_5/2_, respectively, where no Au^3+^ is observed in the analysis. Figure 2d displays the fitting of the resolved C 1s signal where three peaks occur at 284.8, 286.4, and 288.3 eV, respectively, indicative of C–C, C–SH, and O–C=O groups in the MSA capping molecules. The resolved O 1s signal in Figure 2e shows three peaks at 531.6, 533.6, and 528.6 eV, respectively, attributed to C–O, C=O, and Cu–O groups, respectively. Table S1 lists the above analysis results, which indicate the existence of metallic Au, Cu, and Cu_2_O together with the MSA capping molecules.

Raman analysis of the Cu–Au_1.5_NPs (red) is shown in Figure 2f, and the bands at 646, 1251, 1529, 1850, and 2896 cm^–1^ correspond to the stretching vibration of C–S in thiols, the stretching vibration of C–O, the in-plane bending of O–H in the carboxyl group, the stretching vibration of C=O, and the stretching vibration of C–H, respectively, indicating the presence of MSA molecules. As reference, the resonances of the CuNPs (black) are found at 1044, 1526, and 2806 cm^–1^, respectively, assigned to the stretching vibrations of C–O, C–C, and C–H, respectively. As for the AuNCs (blue), the peaks occur at 672, 995, 1499, 1850, and 2823 cm^–1^, respectively, assigned to the stretching vibrations of C–S and C–O, as well as the in-plane bending vibration of O–H in the carboxyl group, the stretching vibration of C=O, and the C–H stretching vibration, indicative of the MSA capping ligands.

FTIR spectroscopy was also used to characterize the Cu–Au_1.5_NPs. In Figure 2g, the band (red) at 3447 cm^–1^ is attributed to the stretching vibration of O–H in carboxylic acid, the 1575 cm^–1^ peak is derived from the stretching vibration of C=O in the carboxyl group, the peak at 1401 cm^–1^ is attributed to the in-plane bending vibration of O–H in the carboxyl group, and the peak at 618 cm^–1^ is the bending vibration of C–H, indicating the existence of MSA. The FTIR spectra of the CuNPs (black) display the peaks at 3433 and 1623 cm^–1^ that are attributed to the stretching vibrations of O–H and C=O in carboxylic acid. The peak at 522 cm^–1^ is assigned to the bending vibration of C–H. The observed FTIR bands for AuNCs are found at 3419 cm^–1^, attributed to the stretching vibration of O–H in carboxylic acid, at 1699 cm^–1^ derived from the stretching vibration of C=O in the carboxyl group, at 1389 cm^–1^ attributed to the in-plane bending vibration of O–H in carboxyl group, and at 639 cm^–1^ assigned to the bending vibration of C–H, indicating the presence of an MSA molecule. The above analytic results demonstrate that the capping agent on the Cu–Au_1.5_NPs is MSA, and amusingly, the PVP molecules also in the solution are not present, which proves no interactions chemically between the PVP and metal-based Cu or Au or Cu–Au nanoparticles.

### 3.2 Electrocatalytic Performance of Cu–AuNPs

**Figure 3:**
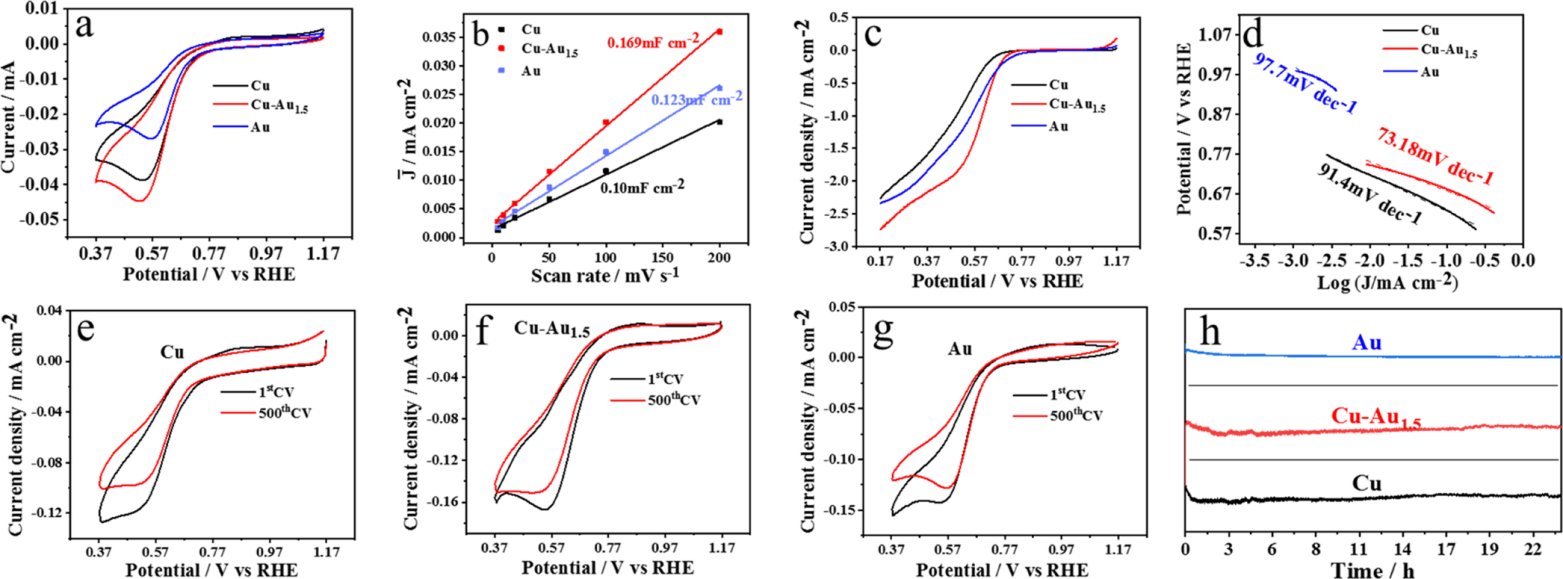
Cyclic voltammogram (CV) plot (a), *C*_dl_ values (b), LSV plot (c), Tafel slopes (d), cyclic durability (e–g), and *i*–*t* chronoamperometric curves (h) of the CuNPs, Cu–Au_1.5_NPs, and AuNCs in O_2_-saturated 0.1 M KOH solutions.

We employed a typical three-electrode configuration to assess the ORR performance. Figure 3a shows the cyclic voltammograms (CV) of the CuNPs, Cu–Au_1.5_NPs, and AuNCs tested in O_2_ saturated with a 0.1 M KOH electrolyte using a scan rate of 10 mV s^–1^. The potential window is set up in the range from 0.365 to 1.165 V vs reversible hydrogen electrodes (RHE). The CuNPs (black), Cu–Au_1.5_NPs (red), and AuNCs (blue) exhibit the onset potentials at 0.697 V, 0.723, and 0.690 V (vs RHE), and the peak potentials appear to be at 0.532, 0.544, and 0.559 V (vs RHE electrode), respectively. However, the peak current densities follow the sequence Cu–Au_1.5_ (−0.045 mA) > Cu (−0.038 mA) > Au (−0.027 mA), which is suggestive of a synergistic effect between the AuNCs and CuNP substrates.

We also investigated the effect of the AuNC loading on the catalytic performance of Cu–AuNPs. Figure S4a shows that Cu–Au_1_ (green) and Cu–Au_2_ (purple) present the onset potential at 0.687 and 0.688 V (vs RHE electrode), and the peak top potentials are reached at 0.515 and 0.508 V (vs RHE electrode), respectively. The peak currents are in the sequence Cu–Au_1.5_ (−0.045 mA) > Cu–Au_1_ (−0.044 mA) > Cu–Au_2_ (−0.04 mA). The results suggest that the ORR catalytic performance of the Cu–Au_1.5_NPs is optimized one.

The electrochemical surface area (ECSA) is an important criterion for evaluating the catalytic activity of electrocatalysts, which can be estimated by the double-layer capacitance (*C*_dl_) value. Figure S5 shows the CV scans in the O_2_-saturated 0.1 mol L^–1^ KOH solutions. The fitting data shown in Figure 3b illustrate that the Cu–Au_1.5_NPs displayed the largest ECSA value, 17.645 m^2^ g^–1^, followed by 14.583 and 12.814 m^2^ g^–1^ for CuNPs and AuNCs, respectively. The ECSA values of Cu–Au_1_ and Cu–Au_2_NPs are measured as 15.979 and 17.396 m^2^ g^–1^, respectively. The ECSA value of the AuNCs is not the largest, which may be attributed to the capping agent MSA (mercaptosuccinic acid).

Figure 3c shows the linear sweep voltammograms (LSV) carried out by using the RDE at a rotation rate of 1600 rpm with a scan rate of 10 mV s^–1^. The half-wave potentials of the CuNPs, Cu–Au_1.5_NPs, and AuNCs are obtained as 0.474, 0.567, and 0.519 V (vs RHE), respectively. Figure 3d shows the Tafel slopes, which are measured as 91.4, 73.18, and 97.7 mV dec^–1^ for CuNPs, Cu–Au_1.5_NPs, and AuNCs, respectively. These suggest that Cu–Au_1.5_NPs are more favorable ones for ORR catalysis.

As a reference, the half-wave potentials of Cu–Au_1_NPs and Cu–Au_2_NPs (Figure S4b) are determined as 0.500 and 0.521 V (vs RHE), respectively, which are larger than Cu–Au_1.5_NPs. The Tafel slopes of the Cu–Au_1_NPs and Cu–Au_2_NPs are measured as 100.5 mV dec^–1^ and 118.2 mV dec^–1^ (Figure S4c), respectively, which are also weaker than Cu–Au_1.5_NPs.

Figure 3e–3g exhibits the durability of the CuNPs, Cu–Au_1.5_NPs, and AuNCs. After 500 CV cycles, the peak current density decreases from −0.263 V to −0.284 V for the CuNPs, and the values for the Cu–Au_1.5_NPs and AuNCs decline from 0.723 and 0.693 V to 0.703 and 0.688 V, respectively. Among them, the Cu–Au_1.5_NPs manifest the largest value and the smallest attenuation. The *i–t* chronoamperometric analyses of the CuNPs, Cu–Au_1.5_NPs, and AuNCs were also performed for 24 h, where the initial voltage was set up at −0.1 V with sampling at an interval of 0.01 s. It can be seen in Figure 3h that the reaction currents decrease in the initial stage for all three samples, and then the current of the AuNCs reaches a plain plateau, while the currents of the CuNPs and Cu–Au_1.5_NPs are gradually restored after about 17 h.

**Figure 4:**
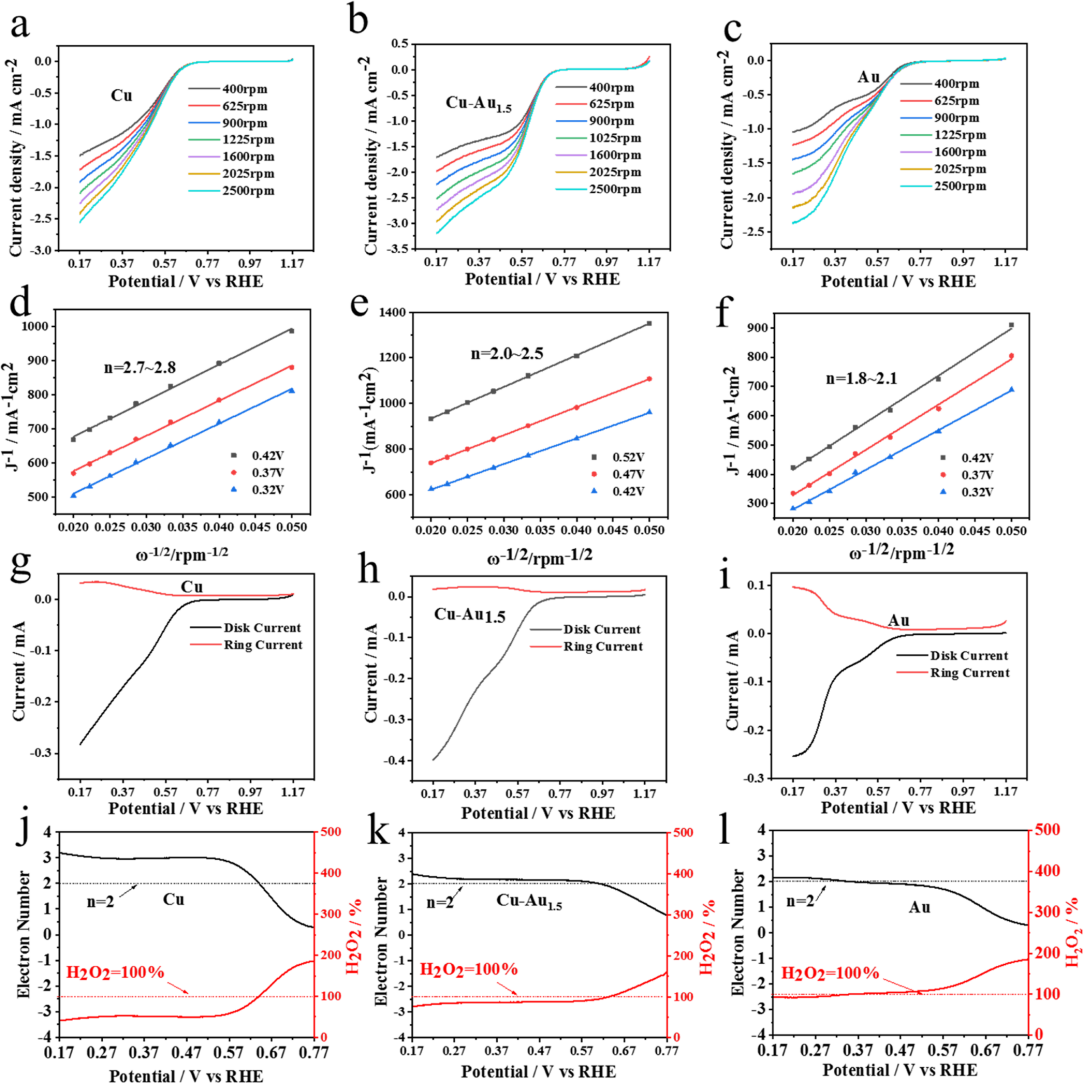
RDE polarization curves (a–c) and K–L plots (d–f) of the CuNPs (a, d), Cu–Au_1.5_NPs (b, e), and AuNCs (c, f). RRDE polarization curves (g–i), the overall electron transfer numbers, and H_2_O_2_ yield rates (j–l) of the CuNPs (g, j), Cu–Au_1.5_NPs (h, k), and AuNCs (i, l).

Figure 4 shows the RDE polarization curves and K–L plots, by which the 2e^–^ selectivity of the CuNPs, Cu–Au_1.5_NPs, and AuNCs is investigated and compared. The potential window was settled between 0.165 and 1.165 V, and the scans started from 1.165 V with a rate of 10 mV s^–1^ in O_2_-saturated 0.1 M KOH solutions. Figure 4a–c shows that the limiting diffusion currents of the CuNPs, Cu–Au_1.5_NPs, and AuNCs increase with the rotation rates. Figure 4d–f shows the K–L plots of the RDE polarization profiles, from which the electron transfer numbers (*n*) are obtained as 2.7–2.8 for the CuNPs, 2.2–2.7 for the Cu–Au_1.5_NPs, and 1.8–2.1 for the AuNCs; meanwhile, the *n* values for the Cu–Au_1_NPs and Cu–Au_2_NPs are 2.2–2.7 and 2.6–2.7, as illustrated in Figure S6, respectively, which means that the Cu–Au_1.5_NPs exhibit the better 2e^–^ ORR selectivity than the CuNPs.

The RRDE analysis was carried out to determine the yield rates of H_2_O_2_ with different electrocatalysts. As illustrated in Figure 4g–4i, the AuNCs exhibit the largest ring/disc current ratio, the CuNPs have the smallest ratio, and the Cu–Au_1.5_NPs manifest a value close to the AuNCs. Figure 4j–l further demonstrates the overall electron transfer number and H_2_O_2_ yield obtained from the RRDE analysis: the ORR electron transfer number of the Cu–Au_1.5_NPs remains very close to 2 for the AuNCs in an O_2_-saturated 0.1 M KOH electrolyte, where the H_2_O_2_ yield at 0.640 V (vs RHE electrode) is up to 95%. Figure S7 shows that the Cu–Au_1_NPs manifest a weaker ORR selectivity than the Cu–Au_1.5_NPs, and the Cu–Au_2_NPs appear closer to the 2e^–^ selectivity of the AuNCs. In comparison, the electron transfer number of the CuNPs is approximately 3, and hence, the H_2_O_2_ yield is the lowest. Table S2 suggests that the Cu–Au_1.5_NP catalyst presents excellent performance on the onset potential and H_2_O_2_ selectivity compared to the previous literature.

The economic cost is an important factor in the selection of the ORR catalysts for H_2_O_2_ production. The price of gold was $62179.54/kg, which is over 7385 times more expensive than that of copper ($8.4237 per kg), according to the official website of Daily Metal Prices on 04 August 2023. The huge price difference makes it meaningful to replace the Au catalyst with Cu–AuNPs, while our studies above also show that the novel Cu–AuNPs confer higher catalytic activity and improved durability than that of the pure AuNCs under the essential conditions of retaining the comparable 2e^–^ ORR selectivity. Thereby, the costs of the Cu–Au_1_, Cu–Au_1.5_, and Cu–Au_2_ bimetallic catalysts were evaluated based on the materials and processing cost as $0.771, $1.156, and $1.541/g, in contrast to the value of the pure AuNCs at a price of $7.708/g. It is clear that the employment of the Cu–Au_1.5_ catalyst for selective H_2_O_2_ production properly balances the cost and electrocatalytic performance.

## 4 Conclusions

This work focused on the characterization and catalytic performance of the Cu–AuNP bimetallic catalysts, which were prepared by a simple galvanic displacement reaction between the surface Cu atoms and Au(Cl_4_)^−^ anions and resulted in the decoration of the CuNPs with the AuNCs. Compared with the pure CuNPs and AuNCs, the optimized Cu–Au_1.5_NPs (*d* = 53.8 nm) exhibited the best catalytic activity and durability with an onset potential of −0.242 V and a Tafel slope of 73.18 mV dec^–1^. Moreover, the Cu–Au_1.5_NPs have a comparable 2e^–^ ORR selectivity to the AuNCs but with a very low price of which the H_2_O_2_ yield at 0.640 V (vs RHE electrode) is up to 95%. This slightly increased cost of the Cu–Au_1.5_NPs compared to pure CuNPs by the bimetallic catalyst’s functionalization enables the production of CuNPs with comparable 2e^–^ ORR selectivity to the pure AuNCs. Furthermore, the Cu–Au_1.5_NPs have higher catalytic activity and durability in alkaline media due to the interaction between the AuNCs and CuNPs, which properly balanced the additional Au cost and a higher ORR catalytic performance. Hence, this offers a better choice of catalyst selection.

## Supporting Information Available

(Figure S1) Schematic illustration of the synthesis of CuNPs and Cu–Au_1.5_NPs; (Figure S2) three-electrode system and modification of the working electrode; (Figure S3) HRTEM image of pure AuNCs; (Figure S4) electrochemical analysis of the Cu, Cu–Au_1_, Cu–Au_1.5_, Cu–Au_2_, and Au catalysts in O_2_-saturated 0.1 M KOH solutions with a scan rate of 10 mV s^–1^: CV curves; RDE polarization curves at 1600 rpm; Tafel plot; (Figure S5) C_dl_ plots of the Cu, Cu–Au_1_, Cu–Au_1.5_, Cu–Au_2_, and Au catalysts in O_2_-saturated 0.1 M KOH solutions and evaluation of *C*_dl_ values by plotting the *J̅* vs scan rate; (Figure S6) RDE polarization curves and K–L plots of the Cu–Au_1_ and Cu–Au_2_ catalysts; (Figure S7) RRDE polarization curves and the overall electron transfer number and H_2_O_2_ yield of the Cu–Au_1_ and Cu–Au_2_ catalysts; (Table S1) XPS analysis of the Cu–Au_1.5_NPs; and (Table S2) latest reports on the electrocatalytic oxygen reduction for H_2_O_2_ production (PDF)
