# Supplementary material for: Functionalized Copper Nanoparticles with Gold Nanoclusters: Part I. Highly Selective Electrosynthesis of Hydrogen Peroxide
Source: ACS Omega. 2023 Sep 22;8(39):36171–8. doi: 10.1021/acsomega.3c03665 (PMC10552511; doi:10.1021/acsomega.3c03665)
Supplement: Supplementary file 1 — ao3c03665_si_001.pdf [file ao3c03665_si_001.pdf]

# Supporting Information

# Functionalised Copper Nanoparticles with Gold Nanoclusters

# Part I. Highly Selective Electrosynthesis of Hydrogen Peroxide ($H_2O_2$)

Kun Luo[1*], Ya Li[1], Tong Liu[1], Xiangqun Zhuge[1], Etelka Chung[2], Andrew Timms[2], Simon P. Graham[3], Guogang Ren[2*]

*1. School of Materials Science and Engineering, Changzhou University, Changzhou 213164, P R China;*

*2. University of Hertfordshire, Hatfield, Hertfordshire, AL10 9AB, UK;*

*3. The Pirbright Institute, Ash Road, Pirbright, Woking, GU24 0NF, UK.*

## 1. Supplementary Figures

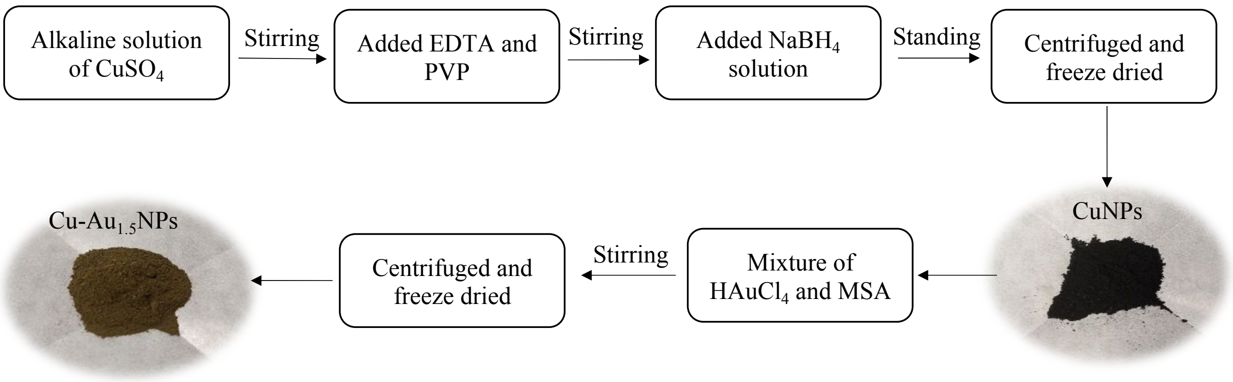


***Fig. S1*** *Schematic illustration on the synthesis of CuNPs and Cu-Au$_{1.5}$NPs.*

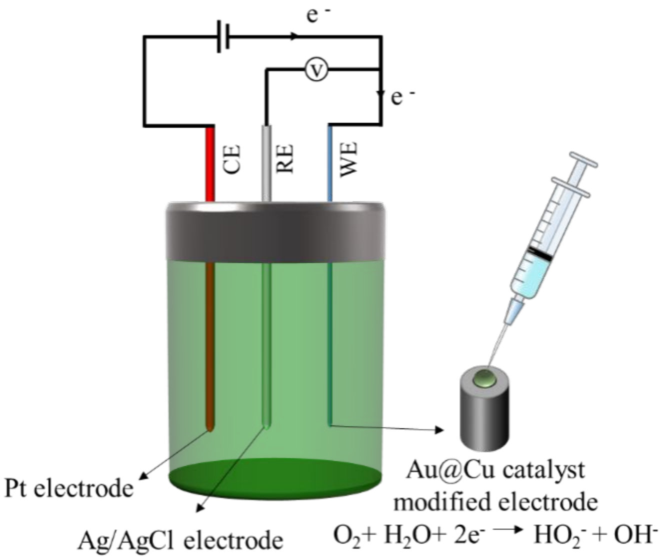


***Fig. S2*** *Three-electrode system and modification of working electrode.*

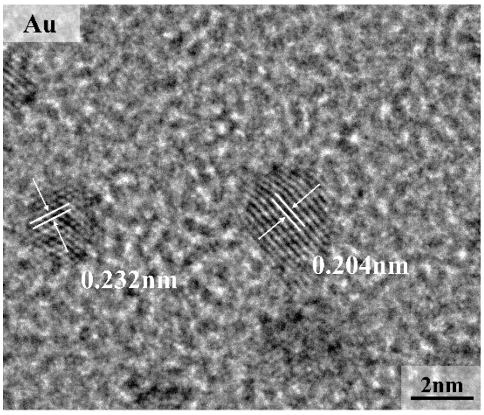


***Fig. S3*** *HRTEM image of pure AuNCs.*

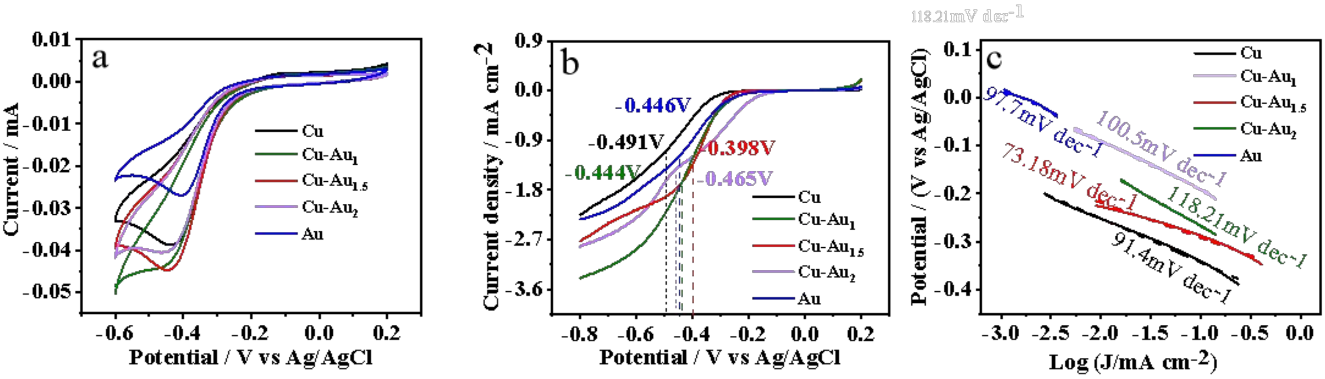


***Fig. S4*** *Electrochemical analysis of the Cu, Cu-Au$_1$, Cu-Au$_{1.5}$, Cu-Au$_2$ and Au catalysts in $O_2$ saturated 0.1 M KOH solutions with a scan rate of 10 mV $s^{-1}$: (a) CV curves; (b) Rotating disk electrode (RDE) polarization curves at 1600 rpm; (c) Tafel plot.*

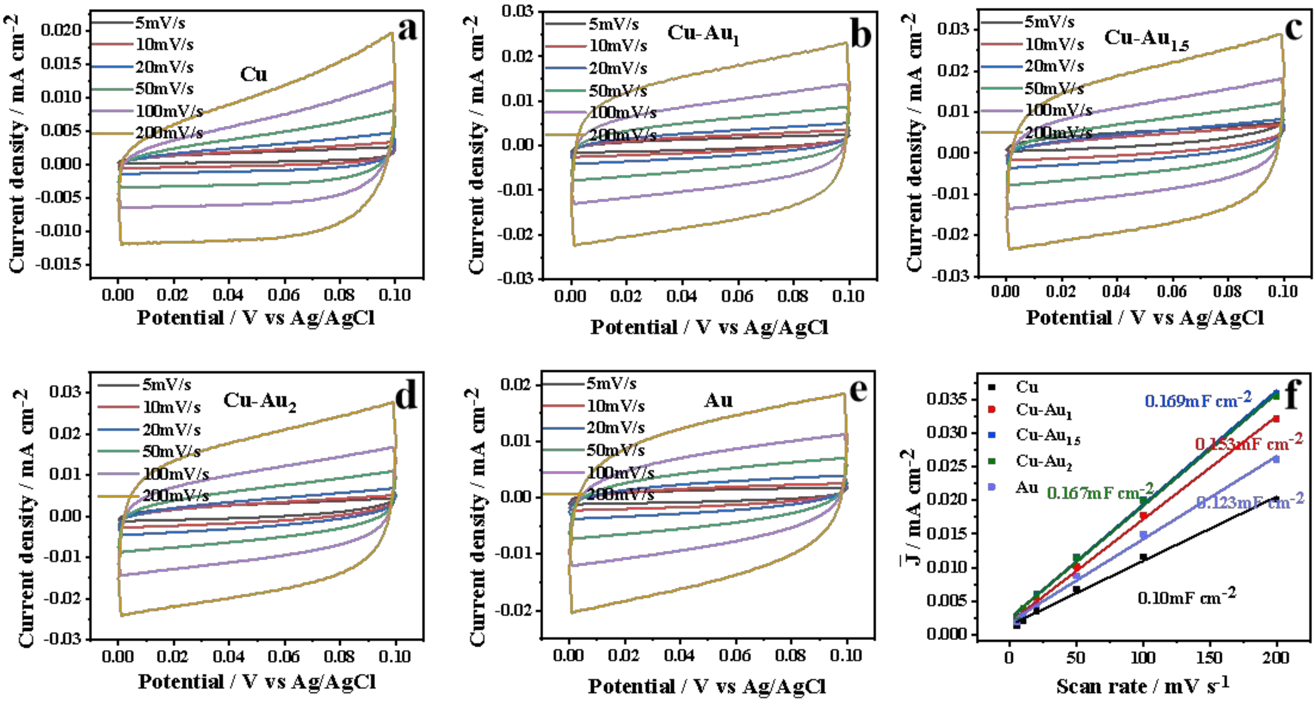


***Fig. S5*** *$C_{dl}$ plots of the Cu (a), Cu-Au$_1$ (b), Cu-Au$_{1.5}$ (c), Cu-Au$_2$ (d), and Au (e) catalysts in $O_2$ saturated 0.1 M KOH solutions and evaluation of $C_{dl}$ values by plotting the $\bar{J}$ vs scan rate (f).*

Electrochemically active area (ECSA) is an important criterion for evaluating the catalytic activity of catalysts, and the double-layer capacitance ($C_{dl}$) value directly reflects ECSA, which is determined by the following formula:

$$\bar{J} = VC_{dl} \qquad (1)$$

$$ECSA = Cdl/Cs \qquad (2)$$

where, *Cs* is the specific capacitance of the electrode with a flat surface, which is found between 20 and 60 μF/cm$^2$, therefore, we can take a modest value of 40 μF/cm$^2$ to calculate the ECSA-based catalysts [1].

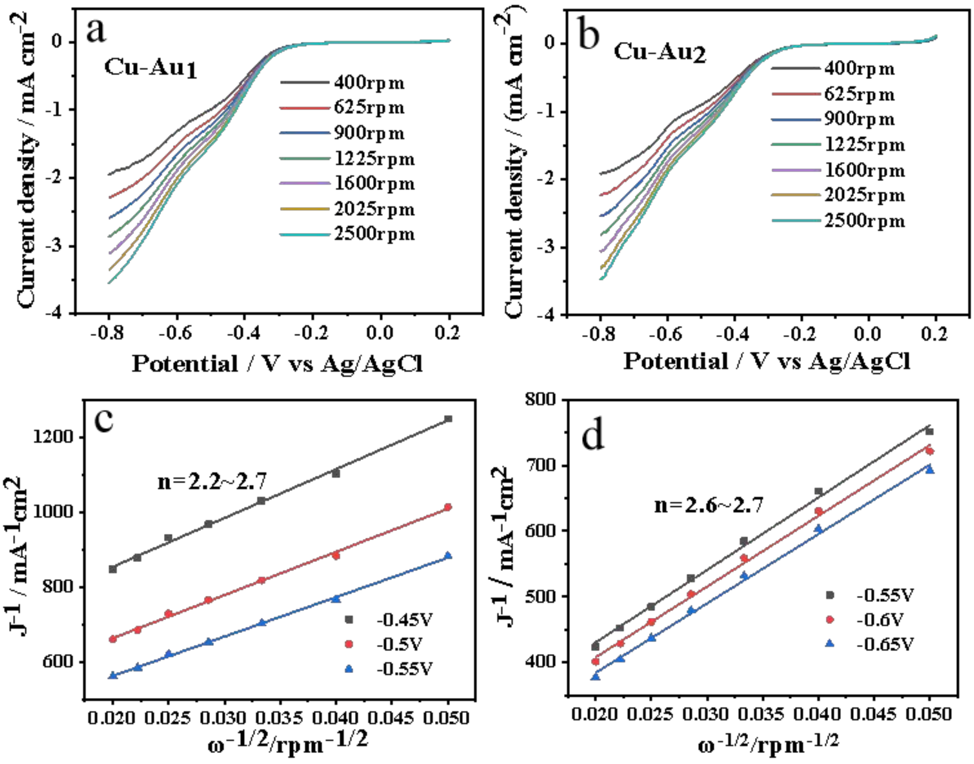


***Fig. S6*** *RDE polarization curves and K-L plots of the Cu-Au$_1$ (a, c) and Cu-Au$_2$ (b, d) catalysts.*

The rotating disk electrode (RDE) polarization curves were tested successively at 400 rpm, 625 rpm, 900 rpm, 1225 rpm, 1600 rpm, 2025 rpm, and 2500 rpm, according to the formula:

$$J^{-1} = J_k^{-1} + J_L^{-1} = J_k^{-1} + \left(B\omega^{\frac{1}{2}}\right)^{-1} \qquad (3)$$

where the inverse of the current density ($J^{-1}$) and the inverse of the square root of the rotational speed ($\omega^{-1/2}$).

The relationship between the two fits the K-L curve corresponding to the RDE polarization curve, and then through the formula Eq.3:

$$B = 0.2nFC_{O_2}D_{O_2}{}^{2/3}\nu^{-1/6} \qquad (4)$$

where *n* is ORR reaction electron number, *F* is Faraday's constant 96487 C moL$^{-1}$, $C_{O_2}$is concentration of oxygen in solution, $D_{O_2}$is diffusion rate of oxygen molecules in electrolyte, $\nu$ is dynamic viscosity of electrolyte.

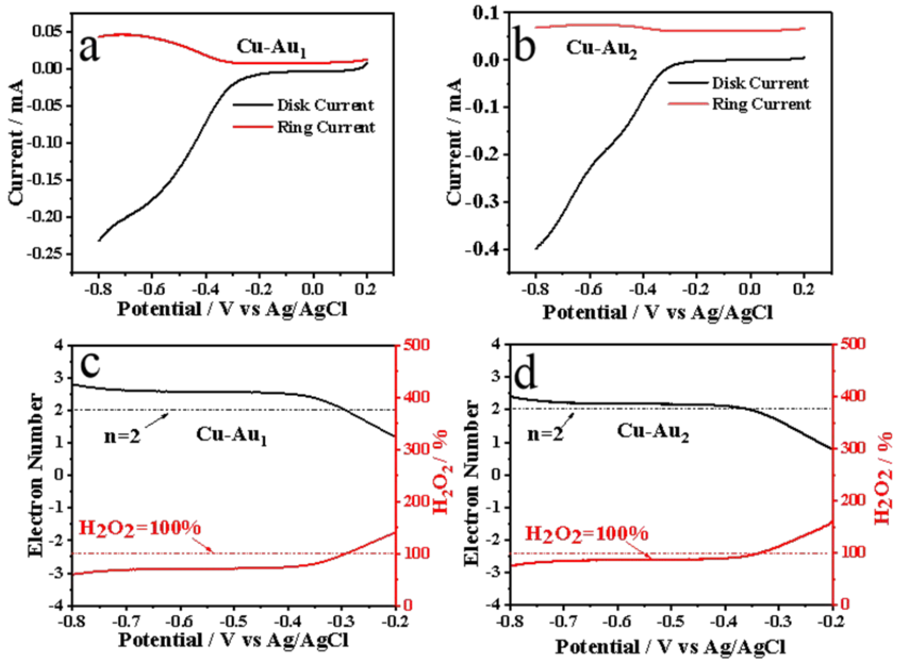


***Fig. S7*** *RRDE polarization curves and the overall electron transfer number and $H_2O_2$ yield of the Cu-Au$_1$ (a, c) and Cu-Au$_2$ (b, d) catalysts.*

The rotating ring-disk electrode (RRDE) test shown that the $H_2O_2$ electrosynthesis of Cu-Au was further investigated by rotating ring disks, and the RRDE polarization curves were obtained, and by formula E-5, E-6 get electron number and $H_2O_2$ yield:

$$HO_2{}^-\% = 2(I_r/N)/(I_d + I_r/N) * 100\% \qquad (5)$$

$$\mathrm{n} = 4I_d/(I_d + I_r/N) \qquad (6)$$

where $I_d$ is the disk electrode current, $I_r$ is the ring electrode current, and $N$ is the current collection efficiency of 0.44.

## 2. Supplementary Tables

***Table S1*** *XPS analysis of the Cu-Au$_{1.5}$ NPs.*

| Elements | Band's components | *BE* (eV) | Atomic percentage (%) | Possible chemistry |
|---|---|---|---|---|
| Au | $Au_{4f7/2}$ | 83.8 | 3.96 | Metallic Au |
| | $Au_{4f5/2}$ | 87.4 | | |
| Cu | $Cu_{2p3/2}$ | 932.6 | 9.09 | $Cu/Cu^{+}$ |
| | | 934.4 | | |
| | $Cu_{2p3/2}$ sat | 939.4 | | $Cu^{2+}$ |
| | | 943.5 | | |
| | $Cu_{2p1/2}$ | 952.6 | | Metallic Cu |
| | | 954.5 | | |
| C | $C_{1s}$ | 284.8 | 54.9 | C-C |
| | | 286.4 | | C-S |
| | | 288.3 | | O-C=O |
| O | $O_{1s}$ | 528.6 | 32.05 | Cu-O |
| | | 531.6 | | O-C |
| | | 533.6 | | O=C |

***Table S2*** Latest reports on the electrocatalytic oxygen reduction for $H_2O_2$ production

| **Catalysts** | **$H_2O_2$ Selectivity [%]** | **Onset potential vs. RHE** | **Electrolytes** | **Refs.** |
|---|---|---|---|---|
| O-CNTs | 90 | 0.8 | 0.1 M KOH | [2] |
| Mo1/OSG-H | 95 | 0.8 | 0.1 M KOH | [3] |
| MOF NSs-300 | 99 | 0.75 | 0.1 M KOH | [4] |
| CB + CTAB | 95.2 | 0.8 | 0.1 M KOH | [5] |
| BN-C1 | 90 | 0.8 | 0.1 M KOH | [6] |
| $Bi_2Te_3$ NPs | 100 | ~0.75 | 0.1 M KOH | [7] |
| Co-POC | 80 | 0.79 | 0.1 M KOH | [8] |
| Co-N-C | ~60 | ~0.82 | 0.1 M KOH | [9] |
| $Au_{25}$ nanoclusters | 86 | 0.8 | 0.1 M KOH | [10] |
| Fe-N-O | 95 | 0.82 | 0.1 M KOH | [11] |
| Cu-Au$_{1.5}$—This work | 95 | 0.71 | 0.1 M KOH | |

**References：**


[1] N. Bhuvanendran, S. Ravichandran, Q. Xu, T. Maiyalagan, H. E. Su, A quick guide to the assessment of key electrochemical performance indicators for the oxygen reduction reaction: A comprehensive review, *International Journal of Hydrogen Energy*, 2022, 47, 11, 7113-7138. DOI: 10.1016/j.ijhydene.2021.12.072.

[2] K. Jiang, S. Back, A. J. Akey, C. Xie, Y. F. Hu, W. T. Liang, D. Schaak, E. Stavitski, J. K. Norskov, S. Siahrostami, H.

T. Wang, Highly selective oxygen reduction to hydrogen peroxide on transition metal single atom coordination, Nature communications, 2019, 10, 1, 1-11. DOI: 10.1038/s41467-019-11992-2.

[3] C. Tang, Y. Jiao, B. Y. Shi, J. N. Liu, Z. H. Xie, X. Chen, Q. Zhang, S. Z. Qiao, Coordination tunes selectivity: two-electron oxygen reduction on high-loading molybdenum single-atom catalysts, Angewandte Chemie, 2020, 132, 23, 9256-9261. DOI: 10.1002/anie.202003842.

[4] M. J. Wang, N. Zhang, Y. G. Feng, Z. W. Hu, Q. Shao, X. Q. Huang, Partially pyrolyzed binary metal–organic framework nanosheets for efficient electrochemical hydrogen peroxide synthesis, Angewandte Chemie, 2020, 132, 34, 14479-14483. DOI: 10.1002/anie.202006422.

[5] K. H. Wu, D. Wang, X. Y. Lu, X. F. Zhang, Z. L. Xie, Y. F. Liu, B. J. Su, J. M. Chen, D. S. Su, W. Qi, S. J. Guo, Highly selective hydrogen peroxide electrosynthesis on carbon: in situ interface engineering with surfactants, Chem, 2020, 6, 6, 1443-1458. DOI: 10.1016/j.chempr.2020.04.002.

[6] S. C. Chen, Z. H. Chen, S. Siahrostami, D. Higgins, D. Nordlund, D. Sokaras, T. R. Kim, Y. Z. Liu, X. Z. Yan, E. Nilsson, Designing boron nitride islands in carbon materials for efficient electrochemical synthesis of hydrogen peroxide, Journal of the American Chemical Society, 2018, 140, 25, 7851-7859. DOI: 10.1039/c4cc01841a.

[7] N. Zhang, F. F. Zheng, B. L. Huang, Y. J. Ji, Q. Shao, Y. Y. Li, X. H. Xiao, X. Q. Huang, Exploring Bi2Te3 nanoplates as versatile catalysts for electrochemical reduction of small molecules, Advanced Materials, 2020, 32, 22, 1906477. DOI: 10.1021/jacs.8b02798.

[8] B. Q. Li, C. X. Zhao, J. N. Liu, Q. Zhang, Electrosynthesis of Hydrogen Peroxide Synergistically Catalyzed by Atomic Co-N-x-C Sites and Oxygen Functional Groups in Noble-Metal-Free Electrocatalysts, Advanced Materials, 2019, 31, 35, 1808173. DOI: 10.1002/adma.201808173.

[9] Y. Y. Sun, L. Silvioli, N. R. Sahraie, W. Ju, J.K. Li, A. Zitolo, S. Li, A. Bagger, L. Arnarson, X. L Wang, T. Moeller, D. Bernsmeier, J. Rossmeisl, F. Jaouen, P. Strasser, Activity-Selectivity Trends in the Electrochemical Production of Hydrogen Peroxide over Single-Site Metal-Nitrogen-Carbon Catalysts, Journal of the American Chemical Society, 2019, 141, 31, 12372-12381. DOI: 10.1021/jacs.9b05576.

[10] Y. Z. Lu, Y. Y. Jiang, X. H. Gao, W. Chen, Charge state-dependent catalytic activity of [Au25(SC12H25)18] nanoclusters for the two-electron reduction of dioxygen to hydrogen peroxide, Chemical Communications, 2014, 50, 62. DOI: 10.1039/c4cc01841a.

[11] N. Humphrey, R. Rodriguez, G. Arias, E. Thai, E. Muro, B. V. Merinov, W. A. Goddard, T. H. Yu, Comparing the oxygen reduction reaction on selectively edge halogen doped graphene from quantum mechanics, Journal of Catalsis, 2020, 381, 295-307. DOI: 10.1016/j.jcat.2019.10.022.
